# Removing Biofilms from Microstructured Titanium *Ex Vivo*: A Novel Approach Using Atmospheric Plasma Technology

**DOI:** 10.1371/journal.pone.0025893

**Published:** 2011-10-10

**Authors:** Stefan Rupf, Ahmad Nour Idlibi, Fuad Al Marrawi, Matthias Hannig, Andreas Schubert, Lutz von Mueller, Wolfgang Spitzer, Henrik Holtmann, Antje Lehmann, Andre Rueppell, Axel Schindler

**Affiliations:** 1 Clinic of Operative Dentistry, Periodontology and Preventive Dentistry, Saarland University Hospital, Homburg/Saar, Germany; 2 Vascular Biology Group, Fraunhofer Institute, Leipzig, Germany; 3 Department of Medical Microbiology, Saarland University Hospital, Homburg/Saar, Germany; 4 Clinic for Oral and Maxillofacial Surgery, University Clinic of Saarland, Homburg/Saar, Germany; 5 Leibniz Institute of Surface Modification (IOM), Leipzig, Germany; 6 Department of Periodontology, Faculty of Dentistry, University of Aleppo, Aleppo, Syria; National Institutes of Health, United States of America

## Abstract

The removal of biofilms from microstructured titanium used for dental implants is a still unresolved challenge. This experimental study investigated disinfection and removal of *in situ* formed biofilms from microstructured titanium using cold atmospheric plasma in combination with air/water spray. Titanium discs (roughness (Ra): 1.96 µm) were exposed to human oral cavities for 24 and 72 hours (n = 149 each) to produce biofilms. Biofilm thickness was determined using confocal laser scanning microscopy (n = 5 each). Plasma treatment of biofilms was carried out *ex vivo* using a microwave-driven pulsed plasma source working at temperatures from 39 to 43°C. Following plasma treatment, one group was air/water spray treated before re-treatment by second plasma pulses. Vital microorganisms on the titanium surfaces were identified by contact culture (Rodac agar plates). Biofilm presence and bacterial viability were quantified by fluorescence microscopy. Morphology of titanium surfaces and attached biofilms was visualized by scanning electron microscopy (SEM). Total protein amounts of biofilms were colorimetrically quantified. Untreated and air/water treated biofilms served as controls. Cold plasma treatment of native biofilms with a mean thickness of 19 µm (24 h) to 91 µm (72 h) covering the microstructure of the titanium surface caused inactivation of biofilm bacteria and significant reduction of protein amounts. Total removal of biofilms, however, required additional application of air/water spray, and a second series of plasma treatment. Importantly, the microstructure of the titanium discs was not altered by plasma treatment. The combination of atmospheric plasma and non-abrasive air/water spray is applicable for complete elimination of oral biofilms from microstructured titanium used for dental implants and may enable new routes for the therapy of periimplant disease.

## Introduction

Plasma jets are ionized local gas flows containing a mixture of charged particles, chemically reactive species and UV radiation which are able to react with biological material or tissues [1]–[5]. They can be generated under normal pressure by means of microwaves, radio frequency (RF) or pulsed direct current (DC) high voltage in so-called plasma jet sources. In medicine, plasma treatment is currently used for blood coagulation, to sterilize surgical instruments and consumables, or to implement hydrophilic properties to surfaces [6]–[9]. Recently, cold atmospheric argon plasma has been used to decrease bacterial accumulation of biofilms, in animal models of wound infection [10] and in chronic wounds of patients in a clinical study [11]. Furthermore, cold atmospheric plasma has been shown to effectively inactivate bacterial biofilms [12].

Biofilms are generated by microbial communities developing on interfaces between solid surfaces and biological fluids. Besides microorganisms they consist of a matrix of exopolysaccharides, proteins and nucleic acids. The development and maturation of oral biofilms are characterized by several stages [13], [14]. Adsorption of salivary proteins, glycoproteins and mucins forms a so called pellicle layer within minutes. Then, planktonic bacteria adhere to the pellicle surface, divide, and recruit additional planktonic cells within minutes to a few hours. A multi-layer biofilm is formed by bacterial growth and co-adherence of further bacteria. The extracellular matrix of the plaque matures as a network of water-soluble and -insoluble glucans that are synthesized by bacterial glycosyltransferases. The established and matured oral biofilm is a three-dimensionally structured community of many microbial species [14] and is relevant for the development of caries and periodontal diseases [13]. Furthermore, biofilms are present on artificial surfaces in the oral cavity such as dentures or implants [15].

The excellent biocompatibility of titanium as an implant material promotes the adsorption of osteoblasts and fibroblasts but also of biomolecular pellicles, and subsequent accumulation of microorganisms on these surfaces [16], [17].

Microbial biofilms may stimulate the induction of inflammatory processes resulting in gingival and surrounding bone inflammation with implant loss [18]. Therefore, methods for the decontamination of titanium surfaces are of great technical and therapeutical interest. Traditional techniques to decontaminate implant surfaces are mechanical scaling, planing and polishing using plastic, Teflon or metal hand curettes as well as ultrasonic systems, commonly in combination with antimicrobial agents [19]–[22]. Furthermore, air abrasion and laser treatment are frequently used for decontamination of implant surfaces [23], [24]. However, due to the microstructured surface of implants the removal of biofilms still poses a special challenge [21], [25]. Mechanical treatment unfortunately causes alterations of the implant surface resulting in a loss of surface micro-texture. Since the surface microstructure of implants is an important parameter for their osseointegration [26], structure-preserving methods are of particular interest. For this purpose, laser decontamination might be a suitable method [23], [24]. A new approach for inactivation of biofilm while preserving the titanium microstructure could be the treatment with cold atmospheric plasma jets, as their antibacterial effect on adherent bacteria and biofilms has been shown in several studies [12], [27]–[31]. The generation of local plasma treatment under atmospheric pressure with low temperatures of around 40°C and the development of small plasma sources make them an attractive candidate for the development of a clinical method for the decontamination of microstructured titanium surfaces [30], [32]. Therefore, the aim of this experimental study was to test the removal of early biofilms formed *in situ* for 24 h and for 72 h by cold atmospheric plasma from the microstructure of sandblasted/etched titanium surfaces commonly used for dental intraosseous implants.

## Results

### Temperature monitoring

During treatment of the microstructured titanium discs, the surface temperature increased instantaneously and reached its maximum in the plasma jet's centre within 5 s. A mean temperature of 43.2°C+/−4.9°C was measured on the titanium surface using a plasma jet power of 5 W. The temperature decreased to values of lower than 40°C in a distance of 2 mm around the centre of the jet. A decrease of the mean surface temperature to 39.1°C+/−3.1°C in the centre of the plasma jet was observed at a plasma jet power of 3 W.

### Plasma treatment of biofilms on titanium discs

**Figure 1:**
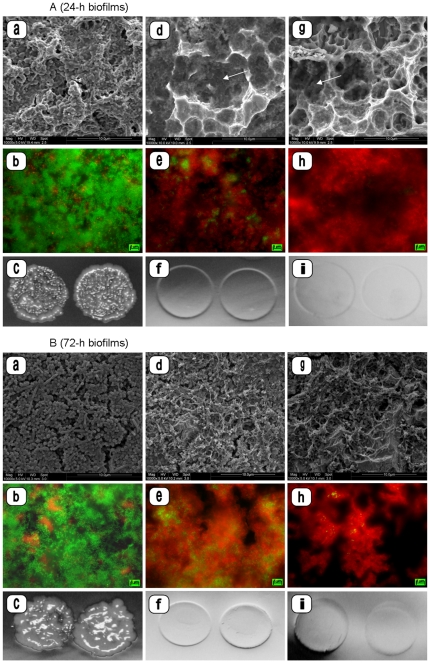
SEM micrographs, fluorescence microscopy images and photographs of contact areas on Rodac plates of untreated (a–c) and plasma treated (d–i) 24-h biofilms (**Figure 1A**) and 72-h biofilms (**Figure 1B**) formed *in situ* on microstructured titanium surfaces (experimental treatment sequence I). Plasma treatment of the biofilms was performed using either a mean plasma jet power of 3 W (d–f) or 5 W (g–i). The untreated titanium surfaces are covered by a dense bacterial biofilm. After plasma treatment biofilm residues are visible in SEM images on the microstructured titanium surfaces (arrow). Higher red fluorescence of biofilms appeared under FM (Magnification: SEM a, d, g: ×10,000; FM b, e, h: ×100). No microbial growth is detectable on Rodac plates after plasma treatment (c, f, i).

**Figure 2:**
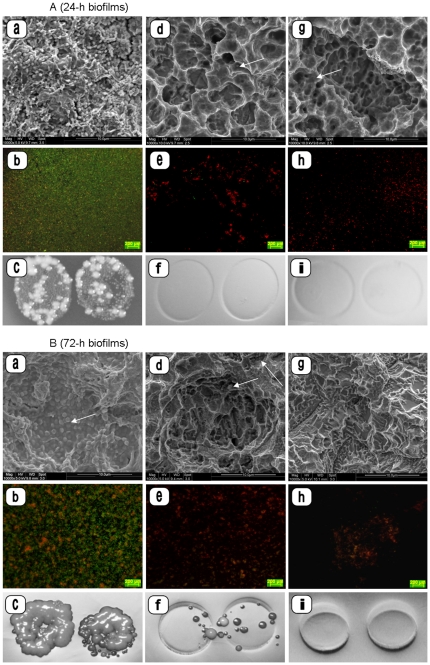
SEM micrographs, fluorescence microscopy images and photographs of contact areas on Rodac plates of 24-h (**Figure 2A**) and 72-h (**Figure 2B**) *in situ* biofilms treated with air/water spray (a–c) or treated with plasma and subsequent air/water spraying (d–i 2 bar, 5 s, 10 mm distance; treatment subgroups II). Plasma treatment of the biofilms was performed using either a mean plasma jet power of 3 W (d–f) or 5 W (g–i). Air/water spraying does not cause biofilm removal (a–c), however air/water spraying after plasma pre-treatment resulted in nearly biofilm free titanium surfaces (d–i). Only sparse biofilm remnants are visible on the microstructured titanium surfaces (arrows). (Magnification: SEM a, d, g: ×10,000; FM b, e, h: ×5). Microbial growth is detectable on Rodac plates after the first cycle of plasma treatment at 3 W (2B-f).

No microbial growth was detected on Rodac plates on any of the 24-h biofilm specimens on titanium discs treated by cold atmospheric plasma (treatment subgroups I–III, Fig. 1A-f, -i, 2A-f, -i, 3A-c, -f, Table 1). In case of the 72-h biofilm samples, on two out of five Rodac culture plates bacterial colonies were detected after the first round of cold plasma at 3 W and subsequent air/water spray treatment (treatment subgroup II-b, Fig. 2B-f). All other Rodac plates displayed no bacterial growth (Table 1, 2).

**Figure 3:**
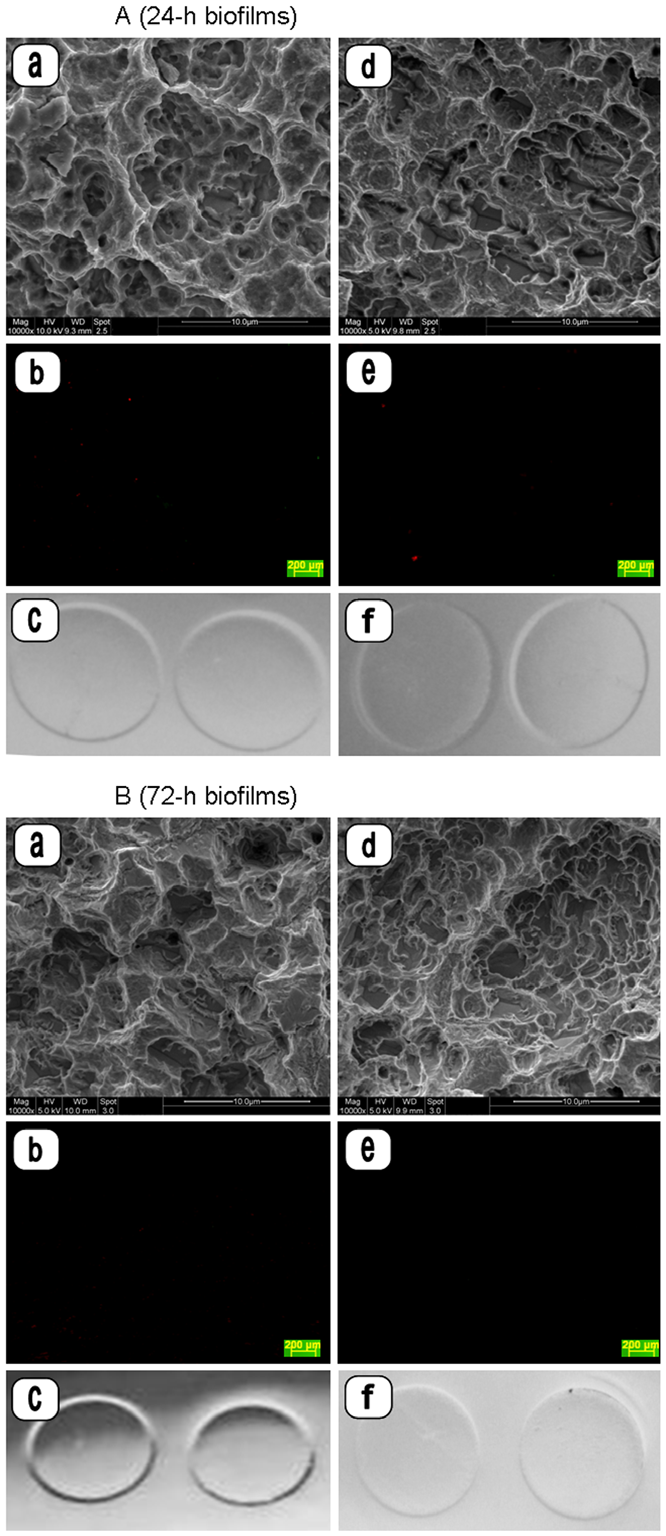
SEM micrographs, fluorescence microscopy images and photographs of contact areas on Rodac plates of 24-h (**Figure 3A**) and 72-h (**Figure 3B**) *in situ* biofilms. Biofilms were pre-treated with plasma ((either 3 W (a–c) or 5 W (d–f)), air/water sprayed, and treated again with plasma (experimental treatment sequence III). No biofilm remnants could be detected on the microstructured titanium surfaces after this sequential treatment. (Magnification: SEM a, d: ×10,000; FM b, e: ×5). No microbial growth is detectable on Rodac plates after plasma treatment (c, f).

**Table 1:** Effects of cold atmospheric plasma treatment on 24-h and on 72-h in situ biofilms formed on 288 microstructured titanium slices.

		Fluorescence microscopy (n = 5/subgroup)				
Treatment subgroups	Positive bacterial cultures(n = 5/subgroup)	total fluorescence median (quartiles)	area without fluorescence, %median (range)	ratio red/green fluorescencemedian	Scanning electron microscopy(n = 3/subgroup)	Protein/sample µg(n = 5/subgroup)
**24-h biofilms (mean thickness 18.8+/−5.7 µm) on microstructured titanium**						
I-a: no treatment	5/5 (100%)	38.7 (20.8/46.1)	2.5 (0.4–8.1)	33.1/84.9	dense biofilm (3/3)	30.0±16.9
I-b: cold plasma 3 W	0/5 (0%)	42.9 (35.9/50.5)	12.6 (4.5–22.5)	110.1/15.6	remnants (3/3)	8.5±5.2*
I-c: cold plasma 5 W	0/5 (0%)	7.8 (2.9/16.3)	26.2 (11.8–62.8)	14.7/0.7	remnants (3/3)	8.2±4.4*
II-a: air/water spray	5/5 (100%)	20.4 (9.9/27.7)	15.3 (4.7–40.9)	29.2/29.3	biofilm (3/3)	17.7±16.3
II-b: cold plasma 3 W+air/water spray	0/5 (0%)	4.7 (1.4/11.3)	88.6 (79.6–97.2)	10.2/0.4	remnants (3/3)	2.9±2.0*
II-c: cold plasma 5 W+air/water spray	0/5 (0%)	1.9 (1.0/5.2)	98.7 (97.0–99.8)	1.7/0.0	remnants (3/3)	2.9±0.7*
III-b: cold plasma 3 W+air/water spray+cold plasma 3 W	0/5 (0%)	1.0 (0.6/1.2)	99.3 (98.7–99.7)	0.2/0.0	no biofilm (2/3)remnants (1/3)	1.3±0.8*
III-c: cold plasma 5 W+air/water spray+cold plasma 5 W	0/5 (0%)	1.0 (0.9/1.2)	99.1 (97.2–99.8)	0.1/0.0	no biofilm (3/3)	1.3±0.9*
**72-h biofilms (mean thickness 91.2+/−18.8 µm) on microstructured titanium**						
I-a: no treatment	5/5 (100%)	57.2 (40.8/67.1)	1.7 (0.0–15.1)	39.9/61.8	dense biofilm (3/3)	60.7±11.6
I-b: cold plasma 3 W	0/5 (0%)	40.8 (31.1/51.0)	14.1 (1.0–24.0)	78.5/26.8	remnants (3/3)	23.7±5.7*
I-c: cold plasma 5 W	0/5 (0%)	37.1 (24.2/40.8)	29.0 (0.5–99.9)	81.0/21.2	remnants (3/3)	17.5±8.8*
II-a: air/water spray	5/5 (100%)	27.5 (18.3/46.0)	19.5 (3.8–67.9)	32.5/35.4	biofilm (3/3)	15.6±3.5
II-b: cold plasma 3 W+air/water spray	2/5 (40%)	6.4 (3.3/10.4)	90.6 (69.4–99.5)	11.2/4.4	remnants (3/3)	3.2±2.4*
II-c: cold plasma 5 W+air/water spray	0/5 (0%)	2.6 (1.5/4.6)	96.7 (80.2–99.5)	4.2/1.9	remnants (3/3)	2.0±2.8*
III-b: cold plasma 3 W+air/water spray+cold plasma 3 W	0/5 (0%)	0.6 (0.1/1.4)	99.8 (95.5/100.0)	0.2/0.1	no biofilm (3/3)	1.5±1.8*
III-c: cold plasma 5 W+air/water spray+cold plasma 5 W	0/5 (0%)	0.0 (0.0/1.1)	99.9 (99.6/100.0)	0.1/0.0	no biofilm (3/3)	1.5±1.4*

In each treatment subgroup 18 independent biofilm covered slices were analyzed by four different investigation methods (144 slices for 24 hours biofilms and 144 slices for 72 hours biofilms). Experimental treatment sequences I–III (no air/water spray, air/water spray, air/water spray plus additional irradiation), subgroups a–c with different intensities of plasma irradiation (no plasma, 3 W, 5 W). Bacterial biofilms were analyzed by contact culture (Rodac), fluorescence microscopy (live/dead staining), scanning electron microscopy (SEM) and analysis of the total protein amounts (bicinchoninic acid protein assay). Fluorescence microscopy was evaluated for three different parameters (total fuorescence intensity, area without fluorescence and ratio of red (dead) versus green (vital) fluorescence.

*statistically significant differences of total protein amounts in comparison to no treatment biofilm control (U-test: p<0.05).

**Table 2:** Effects of plasma treatment on control specimens without biofilms (n = 36, treatment subgroups IV a–c, in triplicates).

		Fluorescence microscopy (n = 3/subgroup)				
Treatment subgroups	Positive bacterial cultures(n = 3/subgroup)	total fluorescence (median)	area without fluorescence (%)	ratio red/green fluorescence (median)	Scanning electron microscopy(n = 3/subgroup)	Protein/sample (µg)(n = 3/subgroup)
**microstructured titanium surfaces without biofilm (Ra: 1.96 µm, Rt: 21,35 µm)**						
IV-a: no treatment	0/3 (0%)	0.1 (0.0/0.7)	99.4 (98.3/99.9)	0.1/0.0	no biofilm (3/3)	<1
IV-b: cold plasma 3 W	0/3 (0%)	0.0(0.0/0.1)	99.8(99.6/99.9)	0.0/0.0	no biofilm (3/3)	<1
IV-c: cold plasma 5 W	0/3 (0%)	0.0 (0.0/0.9)	99.8 (99.7/100.0)	0.0/0.0	no biofilm (3/3)	<1

Fluorescence microscopy (FM) and scanning electron microscopy (SEM) analyses revealed distinct micro-morphological alterations of the adherent microorganisms and the biofilm matrix on the biofilm-covered titanium discs after plasma treatment. A strong decrease of biofilm viability (green fluorescence) and significant reductions in biofilm protein amounts were recorded for both 24- and 72-h biofilms (p = 0.04; treatment subgroups I, Fig. 1, Table 1). Higher mean plasma power (5 W) resulted in extended thinning of the biofilms. However, complete removal of biofilms from the titanium surfaces after the first cycle of plasma treatment could not be observed (Fig. 1, Table 1).

The additional application of air/water spray resulted in further reduction of the biofilm remnants (treatment subgroups II, Fig. 2, Table 1). Strongly reduced red (dead bacterial cells) and green fluorescence (vital bacterial cells) was detected by fluorescence microscopy on titanium discs irradiated with mean plasma powers of 5 W and subsequently air/water sprayed. In addition, the protein amounts were further reduced in comparison to solely plasma treated specimens (p = 0.02). Biofilm covered titanium discs which were plasma irradiated by 3 W and air/water sprayed showed slightly more biofilm remnants in FM and SEM images as compared to samples with 5 W plasma irradiation and subsequent air/water sprayed treatment (Table 1).

**Figure 4:**
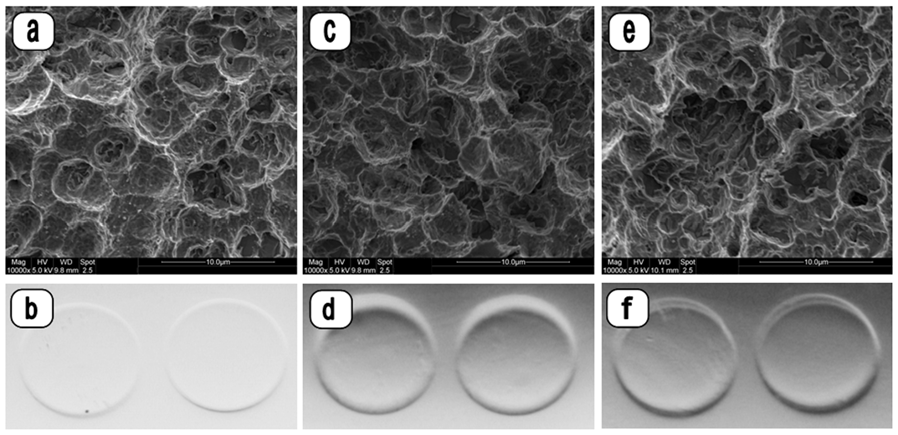
SEM micrographs and photographs of contact areas on Rodac plates (24-h incubation) of untreated (a, b) and plasma treated (c–f) microstructured titanium surfaces without biofilm (experimental treatment sequence IV). Plasma treatment of the titanium surfaces was performed using either a mean plasma jet power of 3 W (c, d) or 5 W (e, f). No surface alterations were detected after plasma treatment of microstructured titanium surfaces. (Magnification: SEM a, c, e: ×10,000). No microbial growth is detectable on Rodac plates after plasma treatment (b, d, f).

A complete removal of biofilm without biofilm remnants were detected after a second cycle of plasma treatment in SEM images in either group of 24- or 72-h biofilm samples treated with a mean plasma power of 3 W and 5 W, respectively, (treatment subgroups III, Fig. 3, Table 1). The microstructured surface of the treated samples was not altered compared to untreated controls (Fig. 4). Residual biofilm bacteria (fluorescence microscopy) and proteins (protein amounts) were completely eradicated (p = 0.02) comparable to the control specimens without biofilms (treatment subgroups IV, Table 2), and no microbial growth was detected by contact cultures on Rodac plates.

### Control biofilm specimens and plasma treatment of titanium discs without biofilms

The biofilm thickness on specimens exposed to the oral cavity for 24 h was 18.8+/−5.7 µm in mean as determined by CLSM. Biofilms exposed for 72 h reached a mean thickness of 91.2+/−18.8 µm. All untreated biofilms presented microbial growth on Rodac plates and showed high viability, represented by green fluorescence, in the fluorescence microscopy images. FM and SEM images indicated complete coverage of titanium discs by biofilms as well as growing of microbes within the microstructured titanium surface. Areas without fluorescence were still present after 24 h of biofilm formation in a range of 0.4 to 8.1% (median: 2.5%). After 72-h biofilm formation the areas without fluorescence ranged from 0 to 15.1% (median: 1.7%). Thorough rinsing of the biofilm covered titanium slices by air/water spray resulted in alterations of the biofilms (SEM), and slightly reduced fluorescence. However, all air/water sprayed biofilms displayed microbial growth on Rodac plates (Fig. 1, 2, Table 1).

Plasma treatment of titanium specimens without biofilms induced no structural alterations of the surface micromorphology (treatment subgroups IV, Table 2, Fig. 4) when using mean plasma powers of 3 W or of 5 W.

## Discussion

The present experimental investigation could clearly show that cold atmospheric plasma is suitable to inactivate and to eliminate early and mature oral biofilms from the microstructure of sandblasted/etched titanium surfaces at acceptable temperatures (<45°C) and without affecting the microstructure of the titanium surfaces. This is in contrast to all other procedures including mechanical cleaning and laser treatment. The microstructure of the titanium specimens used in the present study had a mean roughness of 2 µm with a maximum measured distance between the highest and the lowest level of 30 µm. In the present *in situ* biofilm model the titanium microstructure was almost entirely filled up with biofilm of a mean thickness of 19 µm (24 h) to 91 µm (72 h) which is in accordance with previous observations [22]. Previous data also suggest that 24-h oral biofilms on titanium surfaces are composed by bacteria of distinct diversity which implicates physiological multi-species biofilm characteristics for our model [15]. The 72-h biofilms were even visually detectable after air drying of the surface without optical enlargement and can be assumed as already mature biofilms with strong connectivity. It should also be kept in mind that biofilms on the buccal site of the intraoral appliance were formed against shear-forces of the cheeks, salivary flux from the parotid glands as well as cellular and humoral immune response in the oral cavity. Hence, the biofilms obtained can be regarded stable. As demonstrated by the untreated controls using SEM, FM and CLSM, the biofilms were present within and above the microstructure of the titanium surface and at least the 72-h biofilms completely covered the microstructured surface. As demonstrated by the air/water spray control samples, the biofilms within the titanium microstructure were stable against this high pressure removal approach. The *in situ* biofilm producing volunteers adapted their oral hygiene and nutritional behaviors to create reproducible amounts of biofilms. Oral hygiene measures were carried out without using tooth paste and without brushing of the titanium surfaces. The splints were removed from the oral cavity during drinking. In addition, biofilm inhibiting drinks such as wine or tea were avoided. Since subgingival and anaerobic oral *in situ* biofilms are difficult to reproduce, especially in healthy volunteers, the used oral supragingival biofilm model represents an appropriate approach for testing the potential of cold atmospheric plasma to remove oral biofilms from the microstructure of the titanium surface without destroying the surface morphology.

The very selective destructive power of the plasma jet against these biofilms without morphological destruction of the titanium surface was visualized impressively by the SEM micrographs, and quantified by fluorescence microscopy and colorimetric determination of total protein. Overall, bacterial contact cultures by the Rodac technique underlined the disinfective capacity of the used plasma treatment. Our results confirm previously published data on the efficacy of cold plasma jets for killing of adherent microorganisms or biofilms [27]–[29]. However, a treatment of the biofilm with the plasma jet alone was not sufficient under the chosen conditions to achieve a complete removal of the biofilms. The plaque biofilm was disinfected, was reduced in thickness with disintegration or remnant biofilm structure, and superficial bacteria as well as most bacteria of the deeper biofilm layers were destroyed after plasma treatment alone. The additional application of mechanical cleaning by the air/water spray, however, resulted in an almost complete reduction of the biofilm remnants. The following second plasma treatment cycle performed in our study ensured the complete removal of microorganisms and biofilm remnants even in the microcavities of the microstructured titanium surfaces. To our knowledge, this is the first report of complete inactivation of biofilm bacteria and of complete removal of mature biofilm including extracellular matrix from microstructured surfaces. This was achieved by a sequential approach with cold atmospheric plasma and mechanical cleaning with air/water spray. Undoubtedly, disinfecting and biofilm removing effects of atmospheric plasma powers used in this study might be limited to slim plaque layers. Though, it was possible, to clean surfaces layered with a macroscopically visible thin plaque layer. A complete sterilization and removing of millimeter scaled biofilms may be achieved by a plasma jet at higher performances accompanied with higher temperatures and extended treatment times. However, there are certain limitations concerning the applicability of these parameters under oral conditions, and especially when implant surfaces are treated under *in situ*/*in vivo* conditions. Under clinical conditions however, visible biofilms above the microstructure might also be removed with other implant surface preserving methods like chlorhexidin disinfection followed by spraying, wiping off the soft plaque biofilm with foam rubber pellets or Teflon curettes and softening mineralized plaque by acidic solutions. In the sequence of clinical procedures the plasma treatment could serve as the final approach to remove biofilm remnants from the surface and from the microstructure of titanium surfaces. For all these reasons, we chose experimental parameters simulating realistic conditions to prevent heat-induced damages to the implant surrounding tissues in order to enable a transfer of this method into clinical practice in the near future. The plasma jet was applied in meander-like motion to prevent heating of the titanium discs. The highest temperatures at the titanium surface decreased instantaneously within seconds after the plasma was moved across the surface. The lower mean power setting of the plasma device achieved nearly the same decontamination success as the higher one, suggesting a wide range to establish practically applicable shorter treatment times during surgical open-flap periimplantitis treatment as well as tissue preserving parameter settings. When compared with the untreated reference control specimens, SEM analysis failed to reveal any micromorphologically detectable changes of the microstructured titanium surface. This does not exclude, however, chemico-physical changes of the titanium surfaces due to the formation of the biofilm, or due to the plasma treatment. The plasma application as a result of multiple effects including UV radiation, local temperature increase, jet stream of chemical radicals and electrons leads to an increased hydrophilicity on titanium surfaces [32] as well as an improved bioactivity of plasma treated surfaces [33]. Just as well, effects on the titanium surfaces seem conceivable, which could facilitate the attachment of osteoblasts und thus improve re-osseointegration [32], [34]. Concerning these aspects, the plasma jet source used in the present study appeared to be applicable for removal of biofilms and decontamination of microstructured titanium surfaces. Further investigations are necessary to evaluate the potential of cold atmospheric plasma under *in vivo* conditions and supra- and subgingival biofilms *in situ* as well as their possible role in the clinical setting for an improvement of re-osseointegration of implants. Additionally, plasma treatment could help to disinfect spaces within the implant after removing of the implant-abutment during therapy of periimplant diseases [35]. Although plasma treatment has already been shown in a clinical trial to improve wound healing of the skin [11] the influence of cold atmospheric plasma treatment on oral soft tissues has still to be investigated.

The results of our investigation emphasize the potential of cold plasma jets for antimicrobial applications in dentistry. In particular, the disinfection of biofilm- contaminated microstructured titanium surfaces might be a promising option. In this context, plasma jets could provide a novel approach used in combination with surgical treatment concepts of periimplantitis.

## Materials and Methods

### *In situ* biofilm formation

Overall, 298 titanium discs with biofilms and 36 titanium discs without biofilms were used. The study protocol was approved by the ethical committee of the Saarland Medical Association (vote No. 39/09) and written informed consent was obtained from all participants. Plaque biofilms were formed *in situ* over periods of 24 h and 72 h on 149 microstructured titanium slices each (sand blasted and acid etched, titanium grade 2, Friadent, Mannheim, Germany, 5 mm in diameter, 1 mm in thickness, mean roughness (Ra): 1.96 µm, mean of maximum height of the profile (peak to valley: Rt): 21.35 µm (range: 15.5–31.1 µm), Vickers hardness: 150, tension resistance: 470 N/mm^2^, e-module: 110 GPa). Up to 8 titanium specimens were fixed with silicon impression material (President light body, Coltene, Switzerland) at the buccal sites of the molar and premolar teeth on custom made maxillary splints which were worn by two male volunteers (age: 31 and 43 years). During intraoral exposure, splints were only removed during meals or drinking and meanwhile stored in phosphate buffered saline. Volunteers maintained their normal eating and brushing habits, avoiding biofilm-formation inhibiting drinks such as tea or wine. Neither special cleaning procedures nor agents for chemical plaque control or tooth paste were applied. After exposure in the oral cavity, specimens were rinsed for 10 s with sterile saline solution (0.9%) and the biofilm coverage of the titanium surfaces was checked by stereo light microscopy before further processing of samples.

### Plasma jet treatment

The custom-built (Leibniz Institute of Surface Modification, Leipzig, Germany) non-thermal microwave driven (2.45 GHz) plasma source allowed the adjustment of pulse energy, pulse width and mean power. The plasma source was mounted on a CNC 3-axes linear stage motion system (Steinmeyer MC-G047, Feinmess Dresden GmbH, Germany) to ensure reproducible time, distance and scanning parameters. Treatment was carried out at ambient pressure at a distance of 2 mm between plasma jet nozzle and sample surface. Process gas flow was adjusted at 2.0 l/min helium by mass flow controllers (Bronkhorst, Ruurlo, The Netherlands). The monitored pulse width of the microwave was 5 µs at 250 W (Tektronix TPS2024 oscilloscope, Beaverton, OR, U.S.A.) The mean power of the plasma jet was adjusted by setting of pulse frequency at 3 or 5 W, respectively. The dimensions of the resulting plasma jets were a full width at half maximum (FWHM, near Gaussian profile) of ∼0.5 mm and a jet length of 5 mm. Plasma treatment was carried out in a meander-like scanning mode line by line with a line speed of 1 mm/s and a distance between lines of 0.1 mm. Temperature of the plasma jet was measured dynamically by means of infrared-camera thermography (Optris PI, Optris GmbH, Berlin, Germany) on the surface of titanium discs. Temperature measurements were performed at room temperature with a thermal resolution of ±0.1°C at an optical frame of 160×120 pixels with a frame rate of 100 Hz.

### Treatment sequences and controls

Titanium discs with (24-h and 72-h biofilms, n = 149 specimens each) and without (n = 36) *in situ* biofilms were treated *ex vivo* with cold atmospheric plasma or used as controls. Biofilm thickness was determined using confocal laser scanning microscopy (CLSM, n = 5 for 24-h and 72-h biofilms each). In subgroups of 18 biofilm covered specimens each, 24-h and 72-h biofilm samples were left without any further treatment, irradiated with a mean plasma power of 3 W or with a mean power of 5 W (Table 1, treatment subgroups I), additionally thoroughly rinsed with air/water spray (Table 1, treatment subgroups II, 2 bar, 5 s, 10 mm distance), or rinsed and subjected to a second plasma treatment (Table 1, treatment subgroups III). For the purpose of testing the morphological influence of cold plasma on the etched titanium, samples without biofilms were subjected to plasma treatment at mean microwave powers of 3 W or 5 W, or were kept without any treatment in triplicates (Table 2, treatment subgroups IV).

### Microbiology

Vitality of the microorganisms in the biofilms on eighty titanium discs (n = 40 for 24-h and 72-h biofilms each) was analyzed by contact inoculation of treated and untreated titanium plates with universal brain heart infusion (BHI, Sigma-Aldrich, Taufkirchen, Germany) blood agar using Rodac plates and also by liquid cultures using BHI broth. Nine titanium discs without biofilms served as controls. Following duplicated standardized contact inoculation steps (5 s) of each titanium specimen the biofilm organisms were cultured on Rodac plates (d: 50 mm, Merck, Darmstadt, Germany, Rodac: replicate organism detection and counting imprint technique) at 37°C (5% CO_2_). After contact sampling the titanium surfaces were additionally scraped with a sterile blade and the scraped material was directly transferred into 5 ml liquid medium (LB medium without antibiotics, Sigma-Aldrich, Taufkirchen, Germany) to detect remaining microorganisms. Both, Rodac plates and also liquid cultures were examined at 24 and 48 h. Plates and liquid cultures without microbial growth after 48 h of incubation were assessed to be sterile.

### Fluorescence microscopy (live/dead staining) and confocal laser scanning microscopy (CLSM)

The presence and the viability of biofilms on eighty titanium discs (n = 40 for 24-h and 72-h biofilms each) were assessed by fluorescence microscopy. Biofilms on plasma treated and untreated titanium specimens were stained using a live-dead-staining kit (BacLight Bacterial Viability Kit L7012, Molecular Probes, Carlsbad, USA). Nine titanium discs without biofilms served as controls and were treated accordingly. The live/dead stain was prepared by diluting 1 µl of SYTO 9 and 1 µl of propidium iodide in 1 ml of distilled water. Specimens were placed in 48-well plates, and 100 µl of the reagent mixture were added to each well followed by incubation at room temperature and in the dark for 15 min. Each specimen was carefully positioned on a glass slide covered with mounting oil and stored in a dark space at 4°C until further processing. Samples were evaluated under a reverse light fluorescence microscope (Leitz DMR, Leica, Wetzlar, Germany) equipped with a digital camera (AxioCam MRm Rev. 3, Carl Zeiss Microlmaging, Goettingen, Germany) and according filter sets using the image processing software AxioVision 4.8. (Carl Zeiss Microlmaging, Goettingen, Germany). Images of the titanium surface were captured (5 to 100fold magnification) to estimate biofilm coverage of the specimens. From ten randomly selected sites (420 µm×320 µm) red and green color intensities were separated and medians of red and green fluorescence were calculated using the 0–255 grey scale. Accordingly, total fluorescence median and areas without fluorescence in percentage of surface were calculated using red/green overlays. Continuous data were summarized with median and interquartile ranges (25–75^th^ percentile).

To determine the thickness of biofilms after intraoral exposure of 24 and 72 h, ten titanium specimens with biofilms (n = 5 for 24-h and 72-h biofilms each) were explored by CLSM (confocal laser scanning microscopy; Zeiss LSM 710, Carl Zeiss MicroImaging GmbH, Jena, Germany) after live/dead staining. For this purpose, the biofilms were analyzed using a ×63 oil immersion objective (Plan Apochromat 63×/1.4 Oil; Zeiss). Each biofilm was scanned at four representative areas (rounding the circumference of the titanium slice at distance of 1 mm from the center in 90° steps). Z-series of optical sections were generated by vertical sectioning at 6-µm distances through the biofilm. Image analysis and biofilm thickness measurement were performed using the LSM software ZEN 2008 (Zeiss).

### Detection of total protein

Eighty titanium specimens with biofilms (n = 40 for 24-h and 72-h biofilms each) and nine controls without biofilms were sonicated in 50 µl of RIPA buffer (150 mM NaCl, 1.0% Octylphenyl-polyethylene glycol, 0.5% sodium deoxycholate, 0.1% SDS, 50 mM Tris, pH 8.0, Sigma-Aldrich, Steinheim, Germany), for 10 min at 4°C, and 100 µl ddH_2_O were added. After adding of 150 µl of Micro BCA working reagent, samples were incubated at 55°C for 60 min. Absorbance was measured at 562 nm in a multifunctional microplate reader (Tecan Infinite 200, Magellan V6.6, Tecan, Grödig, Austria) by 10fold determination of each well. Total protein contents of biofilms were colorimetrically estimated (Micro BCA assay, Pierce Biotechnology, Rockford, IL, U.S.A.). Standard protein solutions (albumin) from 0 to 100 µg/ml were used for calibration. Results from plasma treated and/or air/water sprayed specimens were compared with untreated biofilms using the Mann-Whitney-U-test (significance level: p<0.05).

### Scanning electron microscopy (SEM)

Overall 57 titanium discs with (n = 24 for 24-h and 72-h biofilms each) and without biofilms (n = 9) were fixed in glutaraldehyde (2.5% in phosphate buffered saline (PBS); PAA Laboratories GmbH, Pasching, Austria) for 2 h, and rinsed 5 times for 10 min in PBS. Subsequently, the samples were dehydrated in an increasing series of ethanol (50–90% 10 min each; 96% 2×10 min). Finally, the samples were dried in 1,1,1,3,3,3-hexamethyl-disilazane (HMDS, Acros Organics, Geel, Belgium). HMDS was vaporized at room temperature in a clean bench. All samples were mounted on SEM-sample stubs (Plano, Wetzlar, Germany) and sputtered with platinum. SEM analysis was carried out in a FEI XL30 ESEM FEG (FEI Company, Eindhoven, The Netherlands) at magnifications of 1,000 to 10,000fold. The titanium surfaces of each treatment subgroup and both 24-h and 72-h biofilms were scanned for biofilm remnants or bacteria and structural changes caused by plasma treatment in triplicates. If no biofilm or bacteria were detected, titanium surfaces were assumed biofilm free.
